# Integrated Canine-Assisted Services and Art Therapy in Prison: Pilot Study of Animal Well-Being Aspects and Its Impact on Inmate Critical Events

**DOI:** 10.3390/ani16060897

**Published:** 2026-03-13

**Authors:** Susanne Garzillo, Luigi Sacchettino, Luca Esposito, Viviana Orsola Giuliano, Vincenza Panico, Alina Simona Rusu, Rosaria Ponticiello, Alice Nese, Natascia Rizzo, Giuseppe Nese, Francesco Napolitano, Danila d’Angelo

**Affiliations:** 1 Department of Veterinary Medicine and Animal Production, University of Naples Federico II, 81037 Naples, Italy; susannegarzillo@gmail.com (S.G.); vivianaorsola.giuliano@unina.it (V.O.G.); francesco.napolitano3@unina.it (F.N.); 2 Association Zooarteterapia, 81100 Caserta, Italy; 3 Human-Animal Interaction Research Lab, Faculty of Animal Science and Biotechnologies, University of Agricultural Sciences and Veterinary Medicine, 400372 Cluj-Napoca, Romania; alina.rusu@usamvcluj.ro; 4 Caserta Local Health Authority (ASL), 81100 Caserta, Italyalicenese@gmail.com (A.N.); giuseppe.nese@aslcaserta.it (G.N.); 5 CEINGE—Biotecnologie Avanzate Franco Salvatore, 80145 Naples, Italy

**Keywords:** AAS, dog welfare, human–animal relationship, art therapy

## Abstract

Animal-Assisted Services (AAS), together with art therapy, represent an innovative resource in prisons, promoting the emotional and relational well-being of inmates. This pilot study evaluated the effectiveness of an integrated protocol of Animal-Assisted Services and art therapy, with a focus on the selection and monitoring of dogs included in AAS. Forty-two male inmates from a prison in southern Italy were involved. The year-long intervention included weekly group activities with three trained AAS dogs chosen by their personality profiles. The dogs’ well-being was monitored at the beginning, midpoint, and end of the program; data was also collected on the occurrence of critical events for the inmates. The results showed that the dogs maintained a stable profile of psychological and physical well-being and good behavioral regulation, while the inmates showed a significant reduction in critical events. In conclusion, the integrated approach tested, based on the careful selection of dogs, activities tailored to the needs of inmates, and continuous monitoring, proved effective in ensuring animal well-being and promoting improvement in prison conditions.

## Simple Summary

Professional occupations in correctional facilities, such as prisons, present substantial psychological challenges for both personnel and inmates, necessitating innovative interventions for enhancing the institutional socio-emotional climate. This pilot study describes an integrated intervention program that combines canine-assisted treatment with art therapy to promote the well-being of inmates. A significant methodological consideration of our study was to ensure that the dogs involved were not instrumental agents, but active co-participants whose well-being must be guarded before, during and after the program. In line with this, three dogs were selected based on their individual temperaments, and their well-being was longitudinally monitored throughout one-year program. Findings confirmed that the dogs adapted successfully to the prison setting. At the same time, the inmates involved in the program showed a decrease in critical events, such as self-harming behaviors and external incidents. This result substantiates the efficacy of the integrated protocol as a significant asset for rehabilitation, illustrating that favorable outcomes for human participation can be attained without compromising the welfare of the animals involved.

## 1. Introduction

### 1.1. Animal-Assisted Services and the Roles of Included Dogs

In recent decades, Animal-Assisted Services (AAS) have assumed a progressively significant role in therapeutic, rehabilitative, and educational practices, owing to the accumulating scientific evidence of the benefits these interactions confer on the humans and animals involved [1,2,3,4]. Dogs are the most frequently involved non-human animals in AAS contexts. When properly selected for their specific communicative skills, inclination for interspecies collaboration, and behavioral adaptability, these individuals can foster a favorable relational environment, helping to diminish defensive barriers and enhance emotional connection [5]. Recent scientific studies emphasize a methodology for AAS that acknowledges the animal as a “co-therapeutic agent” [6,7,8], aimed at supporting the efforts of professionals engaged in patient care and treatment [9]. The therapeutic benefits of AAS stem from the interspecies interaction, characterized by bidirectional communication, where the animals transcend the tendency to be perceived as mere stimuli or an instrumental agent, assuming the role of reference points with inherent relational, cognitive and emotional capabilities [10]. As a result, AAS seeks to integrate animal welfare as a core methodological and ethical principle within the One Health framework [11,12]. In this context, protecting the animal’s welfare is not just an option, but a fundamental requirement for the ethical and clinical success of the intervention. To ensure that a dog is suitable for AAS, assessing their psychological and emotional stability in different contexts is even more important than assessing their ability to perform specific tasks [7]. This approach reflects the four principles of biomedical ethics: non-maleficence, beneficence, autonomy, and justice, emphasizing the need to implement a “dog-centered” approach [13,14]. Therefore, by prioritizing these values, it is ensured that the animal is a sentient co-participant whose welfare is safeguarded throughout the therapeutic process rather than just an instrumental agent in the therapeutic context. Thus, the identification of suitable dogs for AAS is a crucial phase of any intervention, requiring an assessment of not only the animal’s functional ability in relation to the treatment plan but, more significantly, their personality traits, emotional intelligence, and resilience [15,16]. This stringent, evidence-driven selection process is essential for reducing the risk of fatigue or adverse emotional effects on the animal and for guaranteeing the long-term ethical viability of the ‘co-therapeutic agent’ position. This viewpoint is especially pertinent in high-stress settings like correctional facilities, where the psychological pressures on both humans and animals necessitate heightened ethical scrutiny and methodological rigor in animal selection and program development.

### 1.2. AAS in Prisons in Italy

The incarceration system is considered a dual nature strategy, by combining punishment (to incapacitate offenders and signal deterrence) with rehabilitation (to increase cooperativeness and social functionality), as a way to protect society and address violations of social rules [17]. The prison environment is often perceived by inmates as stressful and detrimental, both physically and psychologically [18]. The imprisonment, characterized by spatial confinement, deprivation of liberty, and the disintegration of social connections, can undermine relational and emotional equilibrium [19], thus fostering the development of latent psychopathological elements and heightening the risk of critical events, i.e., aggressive, self-destructive, or suicidal behaviors [20].

In Italy, this stressful context is reflected in a significant rise in critical events, such as self-inflicted ones (self-harm, attempted suicide, hunger strikes, etc.) and external/others, such as interpersonal aggression, e.g., inmate assaults, assaults on prison workers, collective disturbances, etc. Data from the National Guarantor indicates a generalized escalation in critical events in 2024 compared to 2023, with self-harm rising by 4.1% and attempted suicides surging by 9.3%. Furthermore, a significant increase was observed in aggression and resistance—inmate assaults rose by 7% and assaults on prison workers soared by 22%—as well as collective disturbances [21]. The necessity for implementing initiatives and programs that can enhance the emotional and psychological well-being of inmates is therefore evident. In its contemporary interpretation, and as established by the 1948 Constitution, the primary aims of prison are not merely punishment, but rather re-education, rehabilitation, and social reintegration. This mandate is further supported by the Prison Medicine Reform (Legislative Decree No. 230/1999), which consolidated healthcare resources under the National Health Service. From this viewpoint, structured interventions incorporating Animal-Assisted Activities can serve as a vital therapeutic approach, humanizing punishment within a frequently adversarial environment by fostering rewarding and emotionally significant relationships. AAS elicit resources and emotions in prisoners that are typically repressed to endure the prison setting. This framework underpins the “Riparo, Recupero, ConFido” (Shelter, Recovery, Trust) project, which encompasses the inclusion of animals in correctional settings. This initiative was executed in a correctional facility in southern Italy as a strategy to enhance the efficacy and clinical suitability of prison health services, focusing specifically on inmates that are experiencing considerable psychological distress and assigned to specific high-needs prison sections (Article 32 of Presidential Decree 230/2000). The Individual Therapeutic Treatment Projects were overseen by multidisciplinary teams and concentrated on enhancing self-awareness, refining interpersonal communication and conflict management techniques, and cultivating skills intended to facilitate broader community reintegration.

### 1.3. Art Therapy in Prison

Art therapy is a form of psychotherapy involving artistic expression to enhance the psychosocial characteristics of patients [22]. Art therapy programs in correctional settings have demonstrated efficacy in alleviating symptoms like stress, anxiety, sadness, and hostility, as well as in decreasing recidivism among incarcerated individuals. The existing literature underscores the necessity for research that illustrates the impact of art therapy programs on incarcerated individuals without mental illness diagnoses, especially in elucidating the intermediary variables and methods by which these programs operate. Art therapy prioritizes the establishment of a therapeutic environment defined by perceived safety, reciprocal empathy and creativity through collaborative artistic expression. Women inmates participating in these programs exhibit enhanced capacity to communicate and process intricate emotions, hence fostering higher self-awareness and aiding psychosocial rehabilitation [23]. Art therapy and AAS have both shown their complementary therapeutic roles in facilitating curative and rehabilitative interventions within multidisciplinary settings, as they possess shared mechanisms that enhance social cohesion, foster a sense of belonging, alleviate stress, and aid in emotional regulation [1,24]. Consequently, art expressions and positive interactions with animals share a common denominator as both intrinsically fulfill restorative, trauma-healing, and therapeutic roles. Moreover, both modalities nurture the individual through emotions and feelings that serve as a cohesive modulator of tangible relational connections [25,26]. Therefore, the aim of this pilot study is to investigate the effects of an integrated program—AAS integrated with art therapy—on aspects of the well-being of both inmates and the participating dogs. The following hypotheses were formulated:

- The application of a multiscale selection and monitoring methodology for dogs involved in AAS, based on personality traits, resilience, and behavioral regulation, can monitor potential stress indicators and allow the dog’s well-being profile to be assessed during the intervention period;
- The involvement of inmates in the integrated AAS and art therapy program, which promotes emotional expression and psychosocial regulation, may result in a decrease in the occurrence of critical events during the intervention phase relative to the baseline period.

## 2. Materials and Methods

The pilot study outlines an intervention involving dogs in AAS combined with art therapy, in a novel experimental model of Anthrozoology-Art Therapy (AAS-Art). This initiative took place over one year, from June 2024 to June 2025, at the Federico Uccella prison in Santa Maria Capua Vetere, located in the Campania region, Italy. The project was administratively executed through a complex contract, both in managerial and financial aspects, as stipulated by Article 15 of Law No. 241 of 7 August 1990 (Program agreement for the strengthening of the Sections in accordance with Article 32, Presidential Decree No. 230/2000 and the update to DGRC No. 520 of 13 September 2023; Resolution of the General Director of Local Health Unit (ASL), Caserta No. 18 of 5 January 2024). The interventions were initiated by ASL Caserta—UOC Prison Health Coordination—and by the Regional Directorate for Prison Administration Campania (PRAP) with the aim of “developing Integrated Treatment and Healthcare Activities for prisoners confined in sections pursuant to Article 32 of Presidential Decree 230/2000 who present a condition of significant psychological distress not attributable—exclusively or predominantly—to a psychiatric disorder and who require multidisciplinary care.”

### 2.1. Context of AAS

The AAS-Art project was implemented at the “Francesco Uccella” prison in Santa Maria Capua Vetere, Campania, which comprised seven departments, one of which was female. The prison had a capacity of approximately 826 inmates. The inmates who were designated to the penitentiary section pursuant to art. 32 of Presidential Decree no. 230/2000 and were active in the Danube department of the institute were the recipients of the intervention. The legislation stipulated that prisoners and internees who exhibited behavior that necessitated special caution, as well as for the protection of their companions from potential attacks or oppression, or for whom attacks or oppression by their companions might have been a concern, were assigned to specific institutions or sections where it was more convenient to implement the precautions. The permanence of these placements was verified every six months. The regional regulations integrated national legislation, which previously lacked the operational specifications needed to ensure qualified activities. This integration aimed to organize penitentiary sections to better accommodate appropriate individuals, while respecting several essential preconditions. These included the detection of complex social needs with health relevance through integrated healthcare methods; the exclusion of conditions requiring purely disciplinary or medical management; and the definition of a fixed-term individual Treatment and Therapeutic Project (PTT), which was periodically evaluated through quantifiable indicators.

### 2.2. Ethical Statements, Registrations, and Participant Consent

This pilot study comes to life from the “Riparo, Recupero, ConFido” (Shelter, Recovery, Trust) project and was conducted in compliance with the Declaration of Helsinki, the privacy legislation, and the processing of personal data. The project aims to implement the provisions referred to “in the Agreement of the program for the interventions to strengthen the Sections pursuant to art. 32, Presidential Decree no. 230/2000 and update to DGRC No. 520 of 13 September 2023” incorporated with the Resolution of General Director—ASL Caserta n. 18 of 5 January 2024. Every individual who was involved in this study provided written, informed consent to participate.

The experimental protocols were approved by the Scientific Ethic Committee for Animal Experimentation (Centro Servizi Veterinari-University of Naples Federico II, Italy), reference number: PG/2026/0020813. To guarantee the safety of users and the welfare of animals, the Animal-Assisted Activity was implemented in accordance with the International Association of Organizations for Human-Animal Interaction (IAHAIO) and the Italian National Guidelines for Animal-Assisted Interventions [11,27].

### 2.3. Prison Structure and Organization

The Santa Maria Capua Vetere prison has a regulatory capacity of 826 places. The prison consists of seven wards, six for men and one for women. The wards have indoor and outdoor common areas. In addition to social rooms, some sections have a laundry room and a small gym, two classrooms, a chapel, a vegetable garden that is used in the summer, a tailor’s shop, and two hairdressing salons. Some wards also have social rooms with a communal kitchen, table football, a TV, and a ping pong table. In addition to the green areas, which are also used for visits during the summer months (for both men and women), the outdoor common areas include two sports fields and a gym, which all inmates have access to on a weekly basis. Each section has its own exclusive area for ‘walks’. In terms of healthcare, there is a 24 h medical service and a psychological service for a total of 38 h per week. For daily prison life and ‘dynamic surveillance’, the sections designated for intensive treatment enjoy an open cell regime for 10 h a day with the possibility of 5 h of outdoor exercise per day. All other medium-security sections have cells that are open for 8 h a day [21].

### 2.4. Inmates Enrolled and Eligibility Criteria

The study sample comprised 42 male inmates aged 25 to 45. Among these, 32 were of Italian origin and 10 were international. The sample, in relation to the legal position of the subjects involved in the pilot study, was mixed, including prisoners with definitive, justiciable and recurring convictions; all the subjects involved, however, were recipients of an assignment order to the section pursuant to art. 32 for detected behavioral critical issues (assaults, scuffles, damage to property, offenses against prison police personnel, etc.) not attributable exclusively to psychiatric diagnoses. Participation in the experimental activities was facilitated by a comprehensive interdisciplinary assessment involving personnel from both the prison administration and the health administration (psychologists). The eligibility criteria included the existence of complex social and health demands; the absence of acute symptoms and/or persistent psychopathological decompensation; final sentence and stay in prison for at least the next year; willingness to participate in projects related to animals and art; no allergy to dogs; and no phobia or convictions for animal abuse.

Due to the temporary nature of the assignment under section Ex art. 32, all subjects underwent re-evaluation three and six months after the commencement of project activities, or upon the conclusion of the PTT, also considering the occurrence or absence of the critical events (assaults, altercations, property damage, offenses against prison police personnel, etc.). Monitoring was conducted via psychological interviews with participants, executed by specialized ASL professionals and focused on the examination of the following dimensions: demonstrated motivation for intervention; compliance with activities and attention to regulations; occurrence or non-occurrence of violent behavior; personal introspection and self-awareness.

### 2.5. AAS Dogs

Three dogs were included in the AAS-Art program: Dea, a 2-year-old spayed female Golden Retriever; Polly, a 3-year-old female Irish Setter; and Belka, a 2-year-old spayed female Border Collie. All the dogs worked during the AAS with their dog trainer, who was also their owner. All dogs met the behavioral and physiological requirements of the National Guidelines [9]. In addition, they were well socialized with each other, as they had been part of previous AAS.

#### 2.5.1. Canine Assessment Suitability for AAS-Art Program in a Prison Context

Before the start of the AAS-Art program, the selection was based on an in-depth assessment of each dog’s emotional and behavioral profile, with particular attention paid to their ability to adapt to a complex and sensitive environment such as a prison, their emotional stability, their propensity for socialization and human interaction, and the relationship between the dog and its handler. In particular, the following canine psychometric scales were used by the dog trainers for the selection process:

- Monash Canine Personality Questionnaire (MCPQ) [28] and Positive and negative activation Scale (PANAS) to assess personality and emotional stability [29]. MCPQ assesses the dog’s personality. It is structured around 25 items organized into five main factors: extroversion, amicability, neuroticism, training focus, and motivation. PANAS assesses the dog’s prevailing emotions, distinguishing between positive and negative activation. It is structured into two distinct subscales: positive activation (PA), which encompasses enthusiasm, curiosity, seeking contact and Negative Activation (NA), which encompasses anxiety, avoidance, defensive reactivity. It has been used to assess the suitable profile of dogs for AAS, represented by high PA and low NA, i.e., a proactive, curious but stable and non-anxious dog.
- Canine Frustration Questionnaire (CFQ) [30], Dog Impulsivity Assessment Scale (DIAS) [31], and Lincoln Canine Adaptability and Resilience Scale (L-CARS) [32] to assess the emotional profile and behavioral regulation. CFQ assesses the dog’s response to frustration. It has been used to identify dogs who may exhibit reactive or dysfunctional behaviors in the event of unexpected changes or interruptions that may occur in an AAS setting. It is structured in 21 items, divided into 5 main factors that assess areas related to the impact of frustration in daily life, excitability and motor agitation, negative reactivity, tolerance to failure, and persistence of frustrated behavior. DIAS measures the level of impulsivity. It has been used to assess the frustration of AAS dogs as a risk factor in AAS, where calmness, waiting, self-control, and the ability to manage frustration and multiple stimuli are required. It is structured in 18 items that assess behavioral regulation, aggression and response to novelty, and responsiveness on a Likert scale. L-CARS: measures the dog’s emotional resilience and environmental adaptability, a fundamental characteristic in AAS contexts where the dog is exposed to unpredictable variables. It is structured in 17 items divided into two sections: emotional recovery after stressful situations, adaptability to environmental and social changes.
- Cat/Dog Owner Relationship Scale (C/DORS) to assess the quality of the relationship between dog and owner [33]. C/DORS: assesses the quality of the relationship between owner and dog. In this pilot study, it was used to assess the relationship between the trainer, who was also the owner, and the dog. It is structured into 32 items organized into three main factors that assess interactions: the frequency and quality of shared activities (pet–owner interactions—POI); perceived emotional closeness (PEC) which was measured through emotional bonding and relational satisfaction; and perceived costs (PC) assessed as emotional, time, and financial burdens associated with the dog. The test provides scores ranging from 1 to 5 for each factor. Higher scores indicate greater interaction, greater emotional closeness, or greater perception of costs, respectively.

A certified veterinary behaviorist supervised and validated all data collected by dog trainers to mitigate potential observer bias. The use of standardized psychometric scales (e.g., CFQ, DIAS) further ensured the objective assessment.

#### 2.5.2. Canine Assessment of Well-Being During the AAS-Art Program

In order to monitor well-being throughout the duration of the program, two scales were administered at three different points in time: at the start of the program (T_0_), halfway through the program at 6 months (T_1_), and at the end of the program at 12 months (T_2_). The scales are generally used by veterinarian specialized in AAS—supported by veterinarians specialized in behavioral science—as they follow the “Evaluation of a Dog’s Emotional Disorder scale (EDED)” [34] and “Lincoln Canine Anxiety Scale (LCAS)” [35].

EDED allows for an objective assessment of the dog’s general emotional state and emotional homeostasis. The scale assesses centripetal (self-directed) and centrifugal (towards the environment) behaviors. Centripetal activities are represented by eating, drinking, self-directed behaviors, and sleeping, while centrifugal activities are based on social contact, exploratory skills, and aggression. The scale gives a score according to emotional state: normal: 9 to 12; phobic: 13 to 16; anxious: 17 to 35; mood alterations (dysthymia): 36 to 44.

LCAS allows the detection of signs of anxiety and enables real-time monitoring of the dog’s emotional state during sessions. The main indicators are tremors, vocalizations, hypervigilance, and avoidance or repetitive behaviors. The higher the score, the greater the level of anxiety shown by the dog in that specific situation; the scores are not interpreted based on predefined ranges of normality; instead, they are used as an indicator of change.

### 2.6. The Structure of the AAS Program Integrated with Art Therapy

The program consisted of a group intervention of AAS integrated with art therapy. The group sessions consisted of approximately 15 inmates, operating on a rotating basis. The meetings were held three times a week, lasting about two hours, in the morning and/or afternoon, depending on the organizational needs of the facility. Each dog was involved once a week on a rotating basis to ensure both animal welfare and coverage of all group participants. The setting utilized common areas, designated rooms, and/or large, bright outdoor spaces. Crucially, the most appropriate activities for each AAS dog were chosen based on their individual behavioral characteristics, in strict accordance with the psychometric test results (Section 2.5) and their unique personality traits, ensuring that sessions utilized their strengths and minimized potential stress.

The AAS program was structured into four progressive phases:

- Phase of exposure and acclimatization to the prison setting for both the dogs and the human participants. For example, guided dog observation sessions that emphasized recognizing expressions and calming signals to promote emotional literacy;
- Phase of building interspecies relationships with the inmates, focusing on basic interactions and trust establishment. For example: guided sessions on conscious physical contact, such as grooming sessions tailored to the dog’s individual preferences and tolerance thresholds; exercises in managing proxemic space through loose-leash walking; and scent-based activities (e.g., discrimination tasks) to promote trust and mutual recognition;
- Phase of recreational and leisure activities with inmates, aimed at developing social and emotional skills in a low-pressure environment. For example: joint problem-solving through cognitive activation games where the participant supports the dog’s autonomy; basic cooperative exercises (e.g., “sit”, “stay”); the creation of sensory paths with diverse surfaces; and supervised free interaction (e.g., fetch or tug-of-war) or exploratory walks in designated areas to encourage the dog’s natural behaviors;
- Phase of integrated AAS-Art Therapy sessions: workshops were conducted in the presence of AAS dog through structured activities specifically designed to promote psychosocial rehabilitation in inmates and monitor the emotional well-being of the dog. An example of this is an activity involved using different pictorial and expressive techniques (e.g., drawing, modeling clay) to represent the dog’s personality, the emotions felt during the interaction, or the unique relationship established with the animal, offering a non-verbal channel for emotional processing and self-awareness.

### 2.7. The Multidisciplinary Team

The team consisted of a psychologist/psychotherapist with a postgraduate specialization in interspecies relationships, responsible for planning interventions with inmates; veterinarian specialized in AAS—supported by veterinarians specialized in behavioral science—responsible for supervising and monitoring the dogs’ well-being; three dog trainers with their respective therapy dogs; and an art therapist [36]. Considering the aims of the AAS, the most appropriate activities for each therapy dog were chosen based on their behavioral characteristics, personality traits, and test results. Critical events among inmates were monitored by the Regional Directorate for Prison Administration (PRAP) Campania.

### 2.8. Data Visualization and AI Tools Disclosure

**Figure 1:**
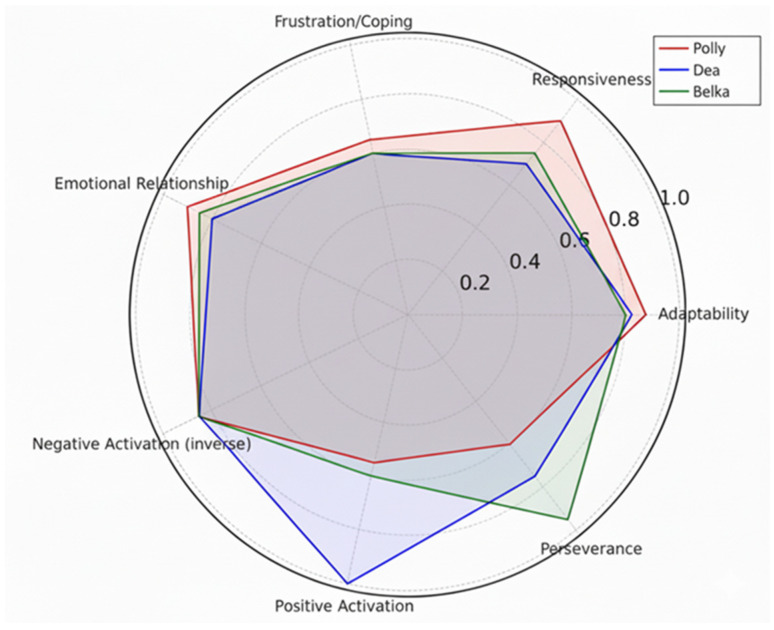
In the radar chart, each axis represents a canine psychological dimension. The value 0 represents the minimum score and the value 1 represents the maximum score; values between 0.3 and 0.8 represent an intermediate score. The results show that Polly is very adaptable and responsive, but less persevering; Dea was found to be the most positive and emotionally stable, but less responsive; Belka was the most persevering and consistent, but slightly less adaptable and with less positive activation.

The graphical representation of Figure 1 was generated with the assistance of ChatGPT (GPT-4o version) (OpenAI, San Francisco, CA, USA). The tool was utilized to facilitate data visualization and to refine the graphical layout, while the underlying raw data were collected and verified solely by the authors. No GenAI tools were used to collect data, design the study, or interpret the results.

## 3. Results

Data for evaluating the suitability and well-being of dogs involved in AAS was obtained using multiscale tools (Section 3.1 and Section 3.2). Moreover, we reported monitoring of the occurrence of critical events in inmates during the sessions in comparison to the baseline period (Section 3.3). Statistical analyses were not conducted in this study due to the exploratory nature of the research and the limited sample size of AAS dogs. Therefore, data are presented in a descriptive way that shows individual behavioral profiles among dogs and longitudinal trends among inmates.

### 3.1. Canine Assessment Suitability for AAS Program in a Prison Context

#### 3.1.1. Results on Emotional Profile and Behavioral Regulation

According to the scores obtained with the CFQ, all the dogs involved fell within normal frustration values (See Table 1): Polly showed lower values of general frustration and unmet expectations, Dea showed average values, while Belka had slightly higher values for unmet expectations.

The scores obtained with the DIAS indicated that all dogs fell within the normal range for impulsiveness and response to novelty: Polly and Belka scored high on factor 3 for responsiveness, and Dea scored in the middle but still maintained responsiveness within normal levels. The data gathered with L-CARS revealed that the three dogs showed good levels of resilience and adaptability. Specifically, Belka scored very high on the perseverance factor, Dea also showed good level of perseverance, while Polly scored a lower level.

#### 3.1.2. Results on General Temperament and Emotional Stability

The data gathered with the PANAS indicated the following aspects: in terms of negative activation, all three dogs showed low scores below the normal range (See Table 2, Figure 1). In terms of positive activation, all three dogs had scores within the normal range. Specifically, Dea showed very high levels of maximum energy and interest; Polly maintained positive scores, with Belka showing slightly more restraint.

In terms of the assessment of the personality style, the results were consistent with the emotional stability results. In particular, Polly was found to have a friendly personality (calm, relaxed); Dea was found to have an extroverted personality (energetic, lively); and finally, Belka was found to have a focus-on-training personality (attentive, intelligent, collaborative).

**Table 2:** This table shows the results on general temperament and emotional stability, carried out to assess the suitability of the three AAS dogs.

**PANAS**	**POLLY**	**DEA**	**BELKA**	**Normal Range**
**Negative Activation**				
OQS Overall Questionnaire Score	0.30	0.29	0.30	0.33–0.63
**Positive Activation**				
OQS Overall Questionnaire Score	0.58	0.68	0.52	0.59–0.85
Factor 1 (Energy & interest)	0.75	1.00	0.85	0.70–1.00
Factor 2 (Persistence)	0.40	0.40	0.45	0.37–0.73
Factor 3 (Excitement)	0.60	0.60	0.70	0.62–0.96
**MPQR**	**POLLY**	**DEA**	**BELKA**	
Personality	**Amicability**	**Extraversion**	**Training focus**	
Characteristic	Easy going	Active	Attentive	
	Friendly	Energetic	Intelligent	
	Relaxed	Lively	Diddable	
	Non-aggressive	Exitable	Obedient	
	Sociable	Hyperactive	Reliable	
		Restless	Trainable	

#### 3.1.3. Results on the Dog–Owner Relationship

In terms of the variables related to pet–owner interactions, the values were near max positive range (See Table 3), with Dea obtaining the maximum score. The scores for perceived emotional closeness were also very high for all three dogs. In particular, the highest scores were recorded for Polly. Finally, the scores for perceived costs were all low in general, with Belka in particular being perceived as the least costly in terms of management.

### 3.2. Canine Assessment of Well-Being During AAS Program

#### 3.2.1. Results of the Lincoln Canine Anxiety Scale

The total behavior scores of LCAS show a clear reduction for all three dogs from time T_0_ to time T_2_. In particular, Polly showed the greatest reduction in percentage terms, with a 60.87% drop in her total score (from 23 to 9). Belka had the largest reduction in absolute terms, with a drop of 15 points (from 28 to 13), corresponding to a percentage reduction of 53.57%. Dea showed a more moderate reduction in her score, with a drop of 9 points (from 25 to 16), equivalent to a reduction of 36%.

#### 3.2.2. Results on Dimensions of the Dog’s Emotional Disorder Scale

**Figure 2:**
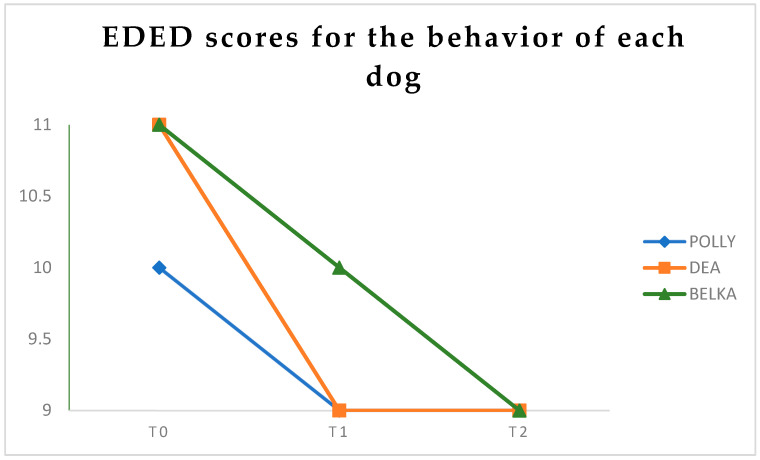
The general behavior patterns of the three dogs during the year-long intervention of AAS-Art. The gradual decrease in signs of stress, observed during the initial acclimatization phase, indicates a functional regulation and adaptive recovery capacity.

The findings indicated that all three dogs consistently diminished stress-related behaviors and exhibited a stable emotional equilibrium (See Figure 2). Polly showed effective emotional regulation, exhibiting a slight decrease in initial stress behaviors from T_0_ to T_2_. The centripetal behaviors of basic functions, including feeding, hydration, and grooming, have consistently exhibited normalcy without significant modifications. At time T_0_, he exhibited hypersomnia, which resolved by time T_1_. The centrifugal behaviors of interactions with the external environment, exploration, and aggression remained unchanged and typical. Social and specific learning also persisted at a functional level. The somatic examination was within normal limits.

Belka exhibited a reduction in stress indicators from time T_0_ to T_2_. The centripetal behaviors of basic functions, including eating, hydration, grooming, and sleep, have consistently remained normal. The centrifugal behaviors of external interactions, particularly in exploratory activity, exhibited initial hypervigilance at To, which subsequently normalized by T_1_. In social learning behavior at T_1_, poor self-control appeared during games, returning at T_2_. On somatic examination, she presented increased emotional urination at T_0_, returning at T_1_.

Dea exhibited an enhancement in stress indicators from period T_0_ to T_2_. The centripetal behaviors of basic functions, including eating, hydration, grooming, and sleep, have consistently remained normal. The centrifugal behavior of external interactions likewise remained constant. During the physical examination, she had heightened emotional urination at T_0_ and reappeared at T_1_.

### 3.3. Results on the Occurrence of Critical Events for the Inmates

**Figure 3:**
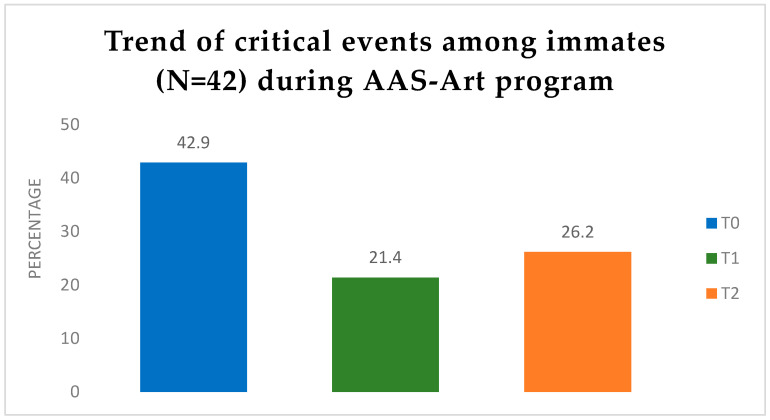
The percentage of inmates for whom critical events are documented by PRAP across the three stages. The data showed that at the start of the AAS-Art program (T_0_), the incidence of critical events was elevated, thereafter decreasing by fifty percent after six months of AAS-Art (T_1_). At the end of the program at 12 months (T_2_), there was a modest increase of approximately 5% relative to T_1_; however, it remained substantially lower than T_0_.

The results showed a general decline in critical events from T_0_ to T_2_, with the AAS phase exhibiting the lowest incidence of these events (see Figure 3). In phase T_0_, 18 of the 42 individuals encountered critical events among inmates. At the T_1_ intervention—six months post-AAS-Art initiation—the incidence of critical events decreased to 9 out of 42 individuals, indicating that critical events had impacted half of the participants at T_0_. At T_2_, there was a marginal rise in the number of participants experiencing critical events compared to T_1_, with 11 out of 42, but the number remained lower than the initial value. Critical events were monitored by the Regional Directorate for Prison Administration Campania.

## 4. Discussion

This pilot study examined the potential effectiveness of a comprehensive selection and monitoring methodology for dogs involved in an integrated Animal-Assisted Services and art therapy program. Character and personality significantly influence a dog’s response to both physical and social environments, including potentially stressful settings such as prisons. Therefore, this study aimed to describe a protocol that emphasizes the dog’s individuality by utilizing standardized tools for evaluation and monitoring within the challenging environment of a correctional facility [37]. All dogs participating in the year-long project were deemed suitable in terms of emotional stability and temperament. Monitoring their well-being showed a positive trend: activities designed and implemented based on each individual’s personality appeared to mitigate potential stressors and foster a favorable attitude among the dogs throughout the study. Prior research concerning the well-being of dogs engaged in AAS has seldom incorporated character and personality evaluations, predominantly concentrating on the examination of physiological and behavioral metrics to discern potential markers of stress or discomfort [6,38]. However, considering that the observed responses are influenced by the dog’s subjectivity, unique character, and personality [39], such metrics may be inadequate to comprehensively depict the animal’s well-being. Incorporating standardized personality and resilience assessments (e.g., PANAS, L-CARS) was crucial for comprehending individual behavior, interpreting atypical reactions, and facilitating their prevention and their monitoring. The analysis of the questionnaires also provided prognostic potential, as the observation of the dogs’ typical behavior in non-working situations allowed for the formulation of reliable hypotheses about their behavior in the working context and the structuring of activities calibrated to their potential. Furthermore, the assessment of the quality of the relationship between the dogs and their trainers was an essential factor for the efficacy of the intervention and the welfare of the dogs [40,41].

### 4.1. Behavioral Stability and Animal Well-Being in a High-Stress Environment

The penitentiary setting inherently imposes significant environmental and social constraints [17,18]. Therefore, CFQ and DIAS allowed us to evaluate the frustration tolerance and impulsiveness of the three dogs, ensuring that the constraints and unfulfilled expectations that are characteristic of this environment did not exceed their coping capacities. Our results suggested that the dogs included in the AAS-Art program exhibited a favorable capacity to regulate frustration and a normal range for impulsiveness and response to novelty. Polly had the lowest levels of general frustration and good coping skills, which showed that she was stable and could handle her emotions in a balanced way. Dea showed average values, highlighting good regulation but at the same time less autonomous control. Belka had slightly higher values for unmet expectations, which means she was more sensitive to problems, but they were still within the normal range [30]. Monitoring how a dog experiences frustration is far more than a technical requirement; in a high-stress setting, a dog’s ability to remain composed when faced with limits or delayed rewards is what prevents impulsive reactions [31]. By selecting dogs with a high capacity for frustration management, human–animal interaction remains a safe, therapeutic space for fragile individuals. Moreover, longitudinal monitoring using the PANAS and L-CARS scales provided a more profound look into the dogs’ emotional lives within the prison [29,32]. The low “negative activation” scores may indicate that the dogs perceived the environment as safe, without triggering states of anxiety or defense. This stability was likely supported by high levels of environmental adaptability and perseverance, particularly in Belka and Polly. These findings suggest that the dogs did more than just “endure” the sessions; rather, their high positive activation scores— Dea’s enthusiasm—seem to suggest that the integrated AAS-Art Therapy protocol acted as a form of social and cognitive enrichment.

As for the canine assessment of well-being during the AAS-Art program, our findings showed that all the dogs progressively reduced their stress-related behaviors, demonstrating good adaptability and resilience. The gradual decrease in signs of stress observed during the initial acclimatization phase may indicate a functional regulation and adaptive recovery capacity. This process was confirmed by the reduction in initial, temporary physiological indicators (e.g., salivation, body shaking). These initial responses could be interpreted as individualized physiological coping strategies in response to a high-stress setting, rather than signs of emotional distress [37]. Furthermore, stable or non-increasing behaviors were specifically linked to individual character traits. For example, passive stress management (hypersomnia in Polly), hyperactive management (uncontrolled play in Belka), and physiological management (emotional urination in Dea) were observed as diverse coping styles based on their personality profiles [7]. Moreover, acknowledging the personality aspects of the dogs involved in AAS represents a crucial step in ensuring their welfare for both the ethical integrity, scientific validity of AAS research, as well as for the psycho-emotional aims of the inmates involved in the program. In particular, Polly, with her calm, thoughtful, introspective, and gentle nature, may have increased awareness of inmates’ behaviors, improved emotional regulation, and facilitated understanding of distance management. Instead Dea, with her extroverted, sociable, and welcoming demeanor, driven by a caring disposition, may have facilitated emotional support, fostered opportunities for positive relational experiences among inmates, and contributed to emotional resilience. Belka, conversely, with her collaborative, empathetic, communicative, and team-oriented disposition, may have facilitated social–emotional learning through group activities, fostering cooperative behaviors and enhancing social skills. Together, they could be experienced as an integrated system of therapeutic resources, in which each dog responds to a particular psychological need, thus fostering the development of a safe space for group interactions (interpersonal and human–animal interactions). The functional adaptation indicated that the selection methodology identified dogs possessing the cognitive flexibility and resilience necessary to navigate challenges in a complex environment, hence safeguarding the well-being of AAS dogs [42]. These data highlight the importance of comprehensive methodological standards in AAS. From this perspective, Sidel and colleagues developed guidelines suggesting standardized procedures, such as behavioral assessment before enrollment, specialized training for researchers, constant stress monitoring, and therapeutic settings aimed at a low impact of stress. In order to further protect the parties involved, the guidelines also called for a conscious informed consent process for both dogs and their owners [43].

### 4.2. Impact on Inmate Emotional Regulation and Reduction of Critical Events

Despite the lack of inferential statistical analysis due to the exploratory nature of this pilot study, the descriptive findings suggested significant interpretations concerning the effect of the AAS-Art program on the inmates’ well-being aspects. The longitudinal monitoring of human’s behavioral data revealed a noteworthy pattern: the implementation of the integrated AAS-Art Therapy protocol seemed to be associated with a decrease in the frequency of critical events among inmates. This change should not be overlooked, considering that inmates often experience traumatic events that tend to recur within the prison environment [18,20]. Indeed, such dynamics may precipitate critical incidents, such as hunger strikes, assaults, self-harm, or suicide attempts, which are the primary symptoms of psychological distress and suffering [19,21]. In particular, our data showed that during the T_1_ phase—six months following the initiation of AAS—the number of participants experiencing critical events decreased to 9 out of 42, thereby indicating that critical events impacted half of the participants who had been affected at T_0_. Although a mild rise was observed at T_2_ (11 out of 42), the overall trend from T_0_ to T_2_ may indicate a positive impact of the intervention on behavioral stability. These descriptive results align with prior research which suggested that inmates engaged in AAS may exhibit various psychosocial benefits—including reduced medication use, decreased violence, lower levels of depression, and enhanced social behavior—while simultaneously increasing personnel safety by improving social skills and decreasing criminal conduct overall [44,45,46,47]. In addition, Fournier’s research demonstrated that inmates participating in such programs showed substantial enhancements across all examined domains [48]. These established findings provide an intriguing framework for interpreting the trend in critical events observed among inmates in our study.

Current scientific studies have indicated that the majority of AAS protocols in prisons predominantly utilize cognitive and performative methodologies, including dog training and professional or educational activities [49,50], while minimally addressing the emotional and social dimensions of the human participants [51,52]. Consequently, individuals with diminished social skills or psychological issues are challenging to engage through conventional methods. Given these pressing concerns, there is a necessity to enhance interventions in prisons not only in a performative manner but also on social and emotional dimensions, as well as to devise novel and more inclusive therapy strategies for all inmates. For this reason, our pilot study introduced a novel experimental model that integrates AAS into art therapy to enhance interventions and create inclusive therapeutic strategies for inmates facing social and psychological challenges. Prior research confirmed the positive impact of art therapy in prisons, particularly in reducing depressive symptoms and enhancing emotional regulation [53,54,55]; in this perspective, our observations suggest that art, facilitated by the dog, could act as a potent medium for communication and social engagement. Therefore, this integrated protocol may serve as a strategy for addressing severe and complex conditions within the prison setting that require targeted interventions.

## 5. Conclusions

In conclusion, our research suggests that a “personality-focused” approach is not simply a theoretical concept, but an essential practice for safeguarding the well-being of dogs included in AAS. By personalizing activities based on each dog’s personality, in this integrated canine-assisted treatment with art therapy, we are able to ensure the animal’s well-being in the challenging prison environment, while simultaneously observing a reduction in critical events in the participating inmates. We do not regard these findings as a definitive conclusion, but rather as proof of concept, considering the explorative nature of this pilot study. Studies involving larger cohorts, as well as non-invasive physiological data collection, are warranted to further investigate these preliminary findings.

## 6. Limitations

Although the results of this pilot study are intriguing, we must consider their practical limitations. Working in a prison environment offers special difficulties that naturally affected this pilot study. For instance, we are fully aware of the small sample size of canine co-participants, as well as of the fact that the analysis of the data is primarily descriptive. We also recognize that without a formal control group, it is difficult to determine for certain that the improvements were due only to the AAS-Art Therapy program, as life in a correctional facility is influenced by many shifting variables. Furthermore, it is important to acknowledge a potential social desirability bias, as some behavioral measures were completed by the dog trainers involved in the program. Although supervised by veterinary behaviorists and based on validated tools, the lack of an independent inter-coder reliability process represents a limitation. Consequently, our preliminary findings represent an exploratory investigation into the efficacy of personalized canine selection (for AAS) and monitoring protocols for mitigating welfare risks in high-stress environments, such as corrections facilities, meriting validation through larger, controlled trials.

## Figures and Tables

**Table 1:** This table shows the results on emotional profile and behavioral regulation, carried out to assess the suitability of the three dogs for AAS.

**DIAS**	**POLLY**	**DEA**	**BELKA**	**Normal Range**
OQS Overall Questionnaire Score	0.43	0.37	0.43	0.42–0.62
FACTOR 1 Behavioral regulation	0.26	0.30	0.36	0.31–0.63
FACTOR 2 Aggression & Response to Novelty	0.36	0.32	0.28	0.22–0.52
FACTOR 3 Responsiveness	0.75	0.52	0.68	0.57–0.83
**CFQ**	**POLLY**	**DEA**	**BELKA**	**Normal Range**
OQS Overall Questionnaire Score	0.33	0.34	0.37	0.33–0.57
PC1 General frustration	0.24	0.28	0.40	0.23–0.53
PC2 Barrier frustration/perseverance	0.35	0.35	0.30	0.37–0.73
PC3 Unmet expectations	0.40	0.50	0.60	0.35–0.69
PC4 Autonomous control	0.32	0.24	0.20	0.24–0.50
PC5 frustration coping	0.40	0.40	0.40	0.30–0.62
**L-CARS**	**POLLY**	**DEA**	**BELKA**	**Normal Range**
Adaptability/Behavioral flexibility	0.87	0.85	0.76	0.72–0.92
Perseverance	0.66	0.80	1.00	0.64–0.98

**Table 3:** The results on dog–owner relationships, carried out to assess the suitability of the three AAS dogs. Higher scores indicate greater interaction and greater emotional closeness between the dog and her trainer.

C/DORS	POLLY	DEA	BELKA	Max Positive Range
Pet–Owner interactions	4.5	5.0	4.4	5
Perceived emotional closeness	4.9	4.5	4.8	5
Perceived costs	4.0	4.3	3.6	5

## Data Availability

The original contributions presented in this study are included in the article. Further inquiries can be directed at the corresponding authors.
